# Health, behaviour and growth performance of Charolais and Limousin bulls fattened on different types of flooring

**DOI:** 10.1017/S175173111900106X

**Published:** 2019-05-07

**Authors:** L. Magrin, F. Gottardo, M. Brscic, B. Contiero, G. Cozzi

**Affiliations:** Department of Animal Medicine, Production and Health, University of Padova, Viale dell’Università 16, 35020 Legnaro, Padova, Italy

**Keywords:** finishing bulls, pen floor, concrete slats, rubber covering, welfare

## Abstract

Intensive fattening of late-maturing breeds on concrete or rubberized slatted floors is the prevalent beef production system in mainland Europe. The rationale behind this study is that specific beef breeds with different slaughter weights might have a diverse response to different flooring systems. The study aimed at assessing whether growth performance, health, behaviour and claw condition of two beef breeds, Charolais (**CH**) and Limousine (**LIM**), would be affected by their housing on concrete (**CS**) or rubber-covered (**RCS**) fully slatted floor. A total of 228 CH (116 on CS; 112 on RCS) and 115 LIM (57 on CS; 58 on RCS) were housed in four and two commercial farms, respectively, in groups of 9.0 ± 2.1 animals/pen with an average space allowance of 3.1 ± 0.2 m^2^. Draining gaps of CS and RCS pens were 16.9 ± 1.7% and 11.6 ± 1.2% of the total surface, respectively. Bulls of both breeds had similar initial body weight (429.4 ± 31.5 kg for CH; 369.6 ± 31.7 kg for LIM), and they were slaughtered when they reached suitable finishing. Charolais had a higher final body weight (**BW**) than LIM (750.8 ± 8.6 *v*. 613.7 ± 10.9 kg; *P* < 0.01), and bulls of both breeds on RCS had higher average daily gain than on CS (1.47 ± 0.02 *v*. 1.39 ± 0.02 kg/day; *P* < 0.05). The percentage of bulls early culled or treated for locomotor disorders were reduced by RCS only for LIM, while RCS tended to prevent the occurrence of bursitis for both breeds. During two 8-h behavioural observations, bulls on RCS performed more head butt/displacements and chases than on CS, and they reduced the frequency of abnormal lying down events. The use of RCS increased mounts’ frequency only in LIM, while its reduced drainage capacity impaired only the cleanliness of CH. *Postmortem* hoof inspection showed longer claw dorsal wall and diagonal lengths, and sharper toe angles for CH on RCS than LIM on both floors. Results of this study point out that fully slatted floors, regardless of being rubberized or not, are not suitable for bulls finished at a final BW above 700 kg due to their detrimental effects on health and welfare. The use of RCS could be recommended as an alternative to CS only if bulls are slaughtered at a lower final BW (around 600 kg), like in the case of LIM breed.

## Implications

Intensive fattening of late-maturing breeds is the predominant beef production system in mainland Europe. These animals are often kept on concrete slatted floor during finishing, but recently, positive effects on their welfare were reported for rubberized slatted floors. However, research on these two flooring solutions has not investigated the response of specific beef cattle breeds with different slaughter weights like Charolais and Limousine. This study suggested that fully slatted concrete floors, bare or rubberized, impaired the health and welfare of heavy bulls like Charolais. Rubberized slats could be a recommended alternative only for lower body weight bulls like Limousine.

## Introduction

The European beef cattle production accounts for almost 8 million tons of meat with France and Germany as main producing countries, followed by United Kingdom and Italy (Hocquette *et al*., 2018). There is a wide diversity among beef producing systems across Europe depending on cattle breeds, feeding and management solutions (European Food Safety Authority (EFSA), 2012). Dual-purpose breeds and crossbreds between dairy cows and late-maturing beef bulls are mainly reared in UK, Ireland and the Scandinavian countries. Late-maturing beef cattle breeds are predominantly fattened in mainland Europe. Young stocks belonging to late-maturing beef breeds are reared in their country of origin (France, Ireland and Easter European countries) for 10 to 14 months of age, and then they are transferred to the country of destination to be finished for 6 to 7 months in specialized fattening units (Gallo *et al*., 2014). Italy imports about 1 million heads per year including young bulls and beef heifers, of which 80% from France (Cozzi, 2007). The fattening of 70%–75% of these imported cattle is mainly carried out in specialized farms located in the Po Valley (Cozzi, 2007). As reported by Gallo *et al*. (2014), Charolais (**CH**) and Limousine (**LIM**) are the most imported French purebreds fattened in Italy. Charolais is generally slaughtered at a heavy BW (over 700 kg), due to its well-known growth potential, feed efficiency and carcass quality (Alberti *et al*., 2008; Clarke *et al*., 2009). Limousine is slaughtered at a lower final weight (on average 590 kg BW) than CH, and it is appreciated by the retail chain for its high dressing percentage and carcass fleshiness (Alberti *et al*., 2008).

Beef farms in Italy operate according to a rather standardized feeding programme in order to promote the maximum daily gain and to guarantee a level of rumination function. In particular, cattle are generally finished using total mixed rations based on maize silage and concentrate feedstuffs (Cozzi, 2007). During finishing, beef cattle are group housed indoors in pens with different types of floor. The fully slatted concrete floor is the most frequent flooring system used in the European fattening units because it requires low space demands and it allows an efficient manure drainage without any bedding material and additional labour for a proper litter renewal to ensure an adequate bull cleanliness (Fallon and Lenehan, 2002). However, concerns about health and welfare of beef cattle kept on fully slatted concrete floors were raised by the EFSA Panel on Animal Health and Welfare (2012). Findings by Platz *et al*. (2007) and Absmanner *et al*. (2009) showed a higher incidence of slipping events and abnormal movements for bulls kept on concrete slatted floors when standing up and lying down than for those housed on alternative flooring solutions like deep litter or different rubber surfaces. Moreover, covering concrete slats with rubber mats improved bulls’ daily gain, claw health and locomotion by decreasing the occurrence of swelling in the leg joints and of white line haemorrhages in the sole (Graunke *et al*., 2011; Elmore *et al*., 2015; Keane *et al*., 2015). However, the scientific research on housing solutions for fattening beef cattle has never investigated the response of specific cattle breeds to different flooring systems. The aim of the present study was to assess whether growth performance, health, behaviour and claw condition of finishing bulls belonging to two beef breeds with different slaughter weight like CH and LIM would be affected by their housing on a concrete or on a rubber-covered slatted floor.

## Materials and methods

### Farms, housing and management

The study was carried out on six commercial beef cattle farms located in the Po Valley, North-eastern Italy. More details on types of floor, housing and management system of the beef cattle farm sample included in this study are described by Brscic *et al*. (2015b), since these farms belonged to the same beef producers’ association that was in charge of cattle feeding and health management.

In each farm, half of the experimental pens had a fully slatted concrete floor (**CS**) with draining gaps of 16.9 ± 1.7 (SD) % of the total surface. In the second half of the experimental pens, concrete slats were covered with 30-mm thick of synthetic rubber (**RCS**) (Riverstick Industries Ltd, Cork, Ireland), designed to match the gap profile of the slats underneath and to allow the drainage of the manure with draining gaps of 11.6 ± 1.2% of the total surface.

A total of 343 finishing beef bulls (228 CH and 115 LIM) were included in the study, and detailed numbers of pens and bulls considered per each farm according to type of floor and breed are reported in Table 1. In all farms, bulls were housed in groups of 9.0 ± 2.1 animals/pen, balanced according to their initial BW (423.5 ± 49.1 and 367.4 ± 17.8 kg BW for CH and LIM, respectively), and the average individual space allowance was 3.1 ± 0.2 m^2^. All pens were equipped with two pressure water bowls for the provision of drinking water. In all farms, bulls were fed a finishing total mixed ratio based on maize silage delivered *ad libitum* once a day in the morning (between 0900 and 1000 h). Dietary samples were collected throughout the study and analysed for DM and CP according to the methods of the Association of Official Analytical Chemists (AOAC, 2000). Analysis of NDF of the same samples was carried out according to Van Soest *et al*. (1991), while starch content was determined by liquid chromatography (AOAC, 2000). Diets offered to CH bulls had an average DM content of 58.6 ± 6.9%, a CP content of 13.5 ± 1.0% DM, a starch content of 31.5 ± 4.4% DM and an NDF content of 31.4 ± 4.4% DM. Diets provided to LIM bulls had an average DM content of 58.5 ± 5.4%, a CP content of 14.0 ± 1.2% DM, a starch content of 32.9 ± 2.4% DM and an NDF content of 29.6 ± 3.7% DM.

**Table 1:** Number of pens and of Charolais and Limousin bulls that were assigned to concrete or rubber-covered slatted floor within breed per each of the six commercial farms

Breed	Charolais			Limousine		
Type of floor	CS	RCS	Total	CS	RCS	Total
Farm A						
Number of pens	4	4	8			
Number of bulls	36	30	66			
Farm B						
Number of pens	4	4	8			
Number of bulls	23	24	47			
Farm C						
Number of pens				3	3	6
Number of bulls				36	36	72
Farm D						
Number of pens	3	3	6			
Number of bulls	30	30	60			
Farm E						
Number of pens	3	3	6			
Number of bulls	27	28	55			
Farm F						
Number of pens				2	2	4
Number of bulls				21	22	43
Total farm sample						
Number of pens	14	14	28	5	5	10
Number of bulls	116	112	228	57	58	115

CS = concrete slatted floor; RCS = rubber covered slatted floor.

### Growth performance, carcass weights and health status

All bulls housed in each pen were weighed as a group in a group livestock scale to reduce stress and risk of injuries at the beginning and at the end of the finishing period. Initial and final BW were used to calculate pen average daily gain (**ADG**). A beef cattle market expert set the end of the finishing period according to the achievement of a suitable finishing of all bulls in each pen. Bull carcass weights were gathered from the slaughterhouse personnel. The same veterinarian belonging to the producers’ association was in charge of bulls’ health in all farms. Individual health status of the animals was daily checked throughout the finishing period. Visually sick/lame animals were temporarily removed from the fattening pens to a sick bay to receive pharmaceutical treatment until healing. The number of bulls that were treated for respiratory and locomotor disorders was recorded as well as the number of bulls that were early culled due to fatal or traumatic events or lameness. A trained fixed veterinarian performed an individual bull’s health check the last month of finishing in all farms. Each bull was visually inspected from the feeding alley, and the occurrence of front and hind leg problems such as bursitis (swelling), alopecia and lesion/wound was recorded as binary variables (presence/absence) according to the Welfare Quality® Assessment protocol for cattle (Welfare Quality®, 2009). The individual cleanliness of the animals, as a sign of comfort around resting, was assessed according to same protocol for cattle.

### Behaviour

In all farms, two 8-h behavioural observation sessions were carried out during the study by a fixed team of four trained assessors. The first observation session was carried out 1 month after the housing of the bulls in the experimental pens, whereas the second one was carried out 2 weeks before the expected slaughter day. Two assessors per each floor type were in charge of the behavioural observations starting right after feed distribution: one assessor recorded the continuous behaviours and the other the events. Position and type of data recorded by each assessor changed in a rotational manner every 2 h to reduce the bias due to the observer effect. The number of bulls standing/lying and eating, ruminating, inactive, resting or involved in other activities in each pen was recorded using the scan-sampling technique with a 5-min interval between two consecutive scans (96 scans/pen/observation session) (Martin and Bateson, 2007). Mounting, chasing, head butt/displacement, slipping, unsuccessful attempts to lie down and abnormal lying down were recorded as events whenever they occurred (1 = occurrence) at pen level using the behaviour-sampling technique (Martin and Bateson, 2007). The ethogram is described in detail by Brscic *et al*. (2015b). A fixed fifth assessor was in charge of measuring durations of the lying-down sequences using a stopwatch in all farms (Welfare Quality®, 2009).

### Postmortem claw measurements

All the animals were slaughtered in the same abattoir owned and managed by the producers’ association. Front and hind feet of a minimum of nine bulls/floor type/farm were randomly chosen and inspected *postmortem* by the same trained veterinarian. Similar to Platz *et al*. (2007), dorsal wall length, diagonal length and toe angle of lateral claws on the left feet and of medial claws on the right feet were measured. A total of 121 bulls were inspected at the slaughterhouse recording front and hind claw measurements (*n* = 480 claw measurements).

### Statistical analysis

Pen was the experimental unit for bulls’ growth performance, continuous behavioural data and events. The single animal was the statistical unit for data regarding carcass weights, time to lying down, clinical traits, cleanliness and claw dimensions. Initial and final BW, ADG, days of fattening and carcass weight were analysed using a mixed model that considered the fixed effect of breed, type of floor and their interaction, with farm nested within breed as random effect and the Bonferroni adjustment option. Continuous behavioural data gathered using the scan-sampling technique were expressed as percentage of bulls performing each behavioural activity per scan per pen, while events were expressed as number of events performed per bull during the observation session at the pen level. These data were processed using a mixed model that considered breed, observation session, type of floor and breed × type of floor interaction as fixed effects, considering farm as a random effect, the observation session as repeated option (Proc Mixed of SAS 9.3; SAS Institute Inc., Cary, NC, USA) and the Bonferroni adjustment option. Intraclass correlation coefficients (**ICC**) were calculated for behavioural data gathered during the two observation sessions to assess agreement between observers *a posteriori* both for breed and type of floor effects using MedCalc Statistical Software (MedCalc Software 17.6 bvba, Ostend, Belgium). Statistical analyses of variables expressed as proportions regarding treated and early culled bulls were performed using *χ* ^2^ tests (with the Marascuilo procedure) to verify their association with the type of floor within breed. Variables gathered as binary regarding bulls’ cleanliness and health were expressed as percentages of bulls. When the prevalence resulted ≥ 1%, they were tested for association with the type of floor within breed using the one-way logistic regression analysis (Proc Logistic of SAS 9.3; SAS Institute Inc., Cary, NC, USA), and the odds ratio (**OR**) and 95% confidence intervals (**CI**) were calculated using RCS as term of comparison. Claw measurements were analysed using a mixed model (Proc Mixed of SAS 9.3; SAS Institute Inc., Cary, NC, USA) that considered the effect of breed, type of floor and their interaction as fixed and of farm as random effects, with the Bonferroni adjustment option. Regarding the normality and homoscedasticity of the errors, the hypotheses of the linear model were graphically assessed by visual inspection of the studentized residuals, and all variables met the model assumptions.

## Results

Regardless of breed and type of floor, bulls had similar initial BW at the onset of the finishing period (Table 2). Final BW of bulls differed according to breed and type of floor (Table 2). In particular, CH bulls were heavier at the end of the fattening period compared to LIM bulls (750.8 ± 8.6 *v*. 613.7 ± 10.9 kg; *P* < 0.01), and bulls kept in RCS pens had higher BW than those kept in CS pens (691.5 ± 8.0 *v*. 673.0 ± 8.0 kg; *P* < 0.05). There were no breed and type of floor effects on the duration of the fattening. Average daily gain tended to be higher for CH than LIM bulls (1.50 ± 0.03 *v*. 1.36 ± 0.03 kg/day; *P* = 0.068), and it was significantly higher for bulls housed on RCS than for those on CS floor (1.47 ± 0.02 *v*. 1.39 ± 0.02 kg/day; *P* < 0.05). Carcass weights of CH bulls were heavier than those of LIM bulls (445.7 ± 6.7 *v*. 385.5 ± 8.2 kg; *P* < 0.5), and it increased for bulls housed on RCS than for those on CS floor (421.9 ± 5.6 *v*. 409.3 ± 5.7 kg; *P* < 0.01). No breed × type of floor interactions were observed for bulls’ growth and slaughter performance (Table 2). Type of floor did not affect the percentage of CH bulls treated for respiratory and locomotor disorders, nor the percentage of CH bulls that were early culled due to traumatic events or lameness (Table 3). In case of LIM, RCS floor significantly reduced the percentage of bulls treated for locomotor disorders and tended to lower the number of early culled bulls (Table 3). Odds ratio values indicated that RCS floor tended to be a preventive measure against the occurrence of bursitis for both breeds (Table 4). The type of floor did not affect the prevalence of CH bulls showing lesion/wound or alopecia. Lesion/wounds were detected in 1.2% of LIM bulls on RCS floor and in none on CS floor. No signs of alopecia were found in LIM bulls housed on both flooring systems. The risk to be dirty was higher for CH bulls kept in RCS pens than for those kept in CS pens, but it did not differ for LIM bulls housed on the same floors (Table 4).

**Table 2:** Growth performance and carcass weights of Charolais and Limousin bulls housed on different types of floor during the finishing period (least squares means) in six commercial farms

Breed (B)	Charolais		Limousine			*P*-value		
Type of floor (TF)	CS	RCS	CS	RCS	SEM	B	TF	B × TF
Number of bulls	116	112	57	58				
Live weight (kg)								
Initial	427.2	431.7	370.5	368.7	32.2	0.313	0.877	0.719
Final	739.6	762.0	606.4	621.1	11.2	0.002	0.029	0.635
Days of fattening	222.5	221.0	179.2	180.4	21.0	0.252	0.969	0.723
Average daily gain (kg/day)	1.46	1.53	1.32	1.40	0.03	0.068	0.022	0.841
Carcass weight (kg)	440.4	451.1	378.2	392.8	7.93	0.011	0.001	0.612

CS = concrete slatted floor; RCS = rubber covered slatted floor; SEM = standard error of mean.

**Table 3:** Effect of the type of floor on the percentage of treated (for locomotor or respiratory disorders) and early culled Charolais and Limousin bulls during the finishing period in six commercial farms

Breed	Charolais		*P*-value	Limousine		*P*-value
Type of floor	CS	RCS		CS	RCS	
Treated bulls (% of bulls)						
For respiratory disorders	4.31	4.46	0.974	10.5	8.62	0.687
For locomotor disorders	4.31	1.79	0.233	15.8	1.72	0.014
Early culled (% of bulls)	6.03	3.57	0.378	3.51	0.00	0.090

CS = concrete slatted floor; RCS = rubber covered slatted floor.

**Table 4:** Effect of the type of floor on the prevalence (%) of Charolais and Limousin bulls with bursitis, lesion/wound, alopecia and dirty coat at the in vivo health check carried out 1 month before the end of the finishing period in six commercial farms

Breed	Charolais				Limousine			
Type of floor	CS	RCS	OR (95% CI)^1^	*P*-value	CS	RCS	OR (95% CI)^1^	*P*-value
Bursitis	22.5	13.5	1.87 (0.90 to 3.88)	0.092	34.5	19.0	2.26 (0.95 to 5.33)	0.064
Lesion/wound	7.8	2.9	2.87 (0.74 to 11.1)	0.128	0.0	1.2	–	–
Alopecia	9.8	8.6	1.15 (0.45 to 2.95)	0.776	0.0	0.0	–	–
Dirtiness	43.5	67.6	0.37 (0.25 to 0.55)	<0.001	37.0	44.8	0.724 (0.42 to 1.24)	0.237

CS = concrete slatted floor; RCS = rubber covered slatted floor.

1 Estimated odd ratios (OR) and 95% confidence intervals (CI) using RCS within breed as term of comparison.

**Figure 1:**
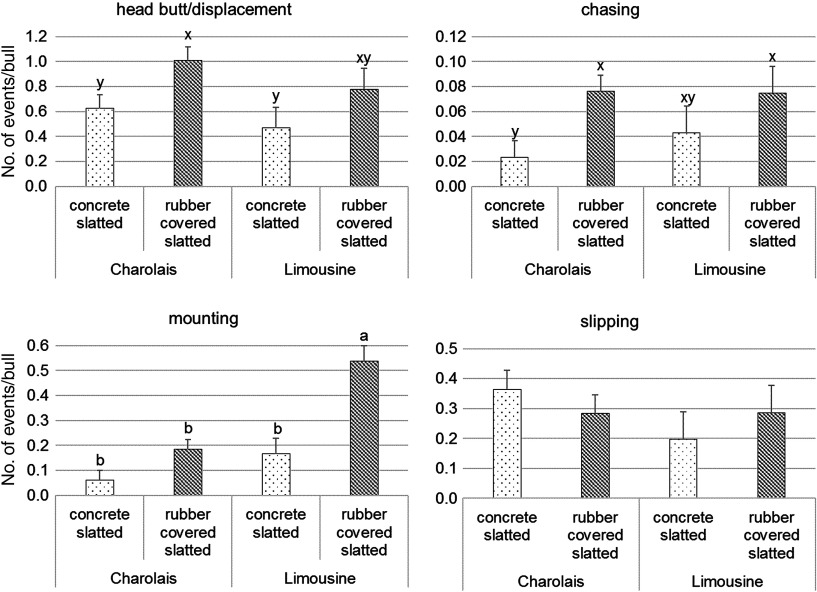
Effect of the type of floor × breed interaction on the number of events performed by bulls during the 8-h observation sessions (least squares means) in six commercial farms. Different letters indicate significant differences within a given event (a,b: *P* < 0.05; x,y: *P* < 0.10).

**Figure 2:**
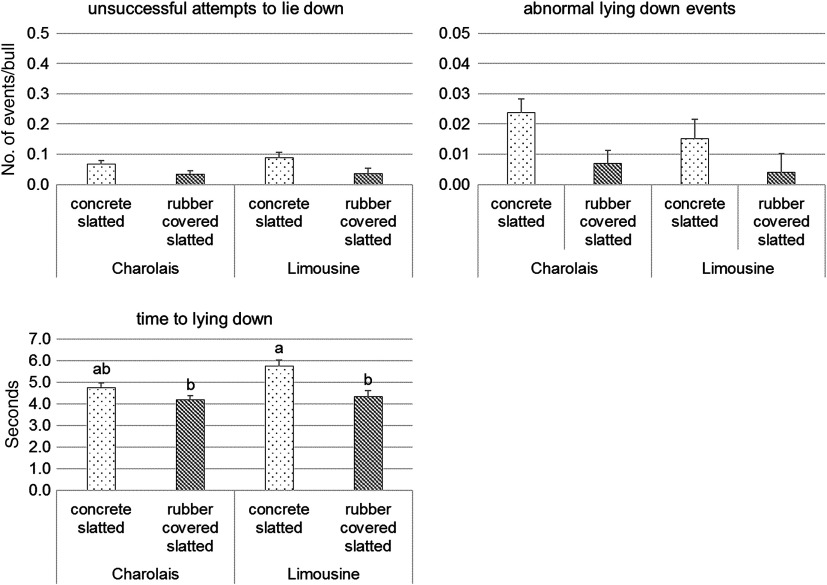
Effect of the type of floor × breed interaction on the lying-down behaviour of bulls during the 8-h observation sessions (least squares means) in six commercial farms. Different letters indicate significant differences within a given lying-down behaviour for *P* < 0.05.

During the two observation sessions the agreement between observers was good both for breed and for the type of floor, since the ICC calculated on the percentages of all behavioural variables were ≥0.70. Observation of continuous behaviour showed a similar proportion of CH and LIM bulls standing, and there were no type of floor and observation session effects on standing behaviour (Table 5). Sternal recumbency with all four limbs folded underneath the body was the most frequent lying postures of all bulls, and its recorded frequency did not vary between types of floor, nor between breeds. It was affected by the observation session, being more frequently at the beginning of the fattening than at the end of it (38.5 ± 2.4 *v*. 30.3 ± 2.4%; *P* < 0.05). The proportion of bulls lying with one front limb extended was higher for CH than LIM bulls (18.2 ± 0.9 *v*. 13.0 ± 1.5%; *P* < 0.05), and for bulls housed on CS than for those housed on RCS floor (20.0 ± 1.2 *v*. 11.1 ± 1.3%; *P* < 0.01). Breed and type of floor had no effect on the proportion of bulls lying with two limbs extended or with lateral recumbency, or resting. Among them, only the proportion of bulls with lateral recumbency varied for the observation session effect, being more frequently at the end than at the beginning of the fattening (3.77 ± 0.9 *v*. 7.09 ± 0.9%; *P* < 0.05). Both breeds performed eating and ruminating activities with a similar frequency, and the type of floor did not affect them (Table 5). Bulls performed rumination predominantly during lying, and more frequently at the beginning of the fattening (15.9 ± 1.4 *v*. 8.79 ± 1.4% for the first and second observation, respectively; *P* < 0.01). Breed × type of floor interactions for the occurrence of events are shown in Figure 1. A significant type of floor effect (*P* < 0.05) was observed for head butt/displacement and chasing events that were recorded with a higher frequency in bulls of both breeds on RCS compared to CS floor (0.89 ± 0.10 *v*. 0.55 ± 0.10 for head butts/displacements; 0.08 ± 0.01 *v*. 0.03 ± 0.01 for chases). The use of RCS floor increased the frequency of mounting events only in LIM bulls while slipping events did not differ according to both breed and type of floor. Regardless of breed, bulls kept in CS pens had a higher frequency of unsuccessful lie-down attempts (0.08 ± 0.01 *v*. 0.03 ± 0.01; *P* < 0.05) and of abnormal lie-down events (0.02 ± 0.004 *v*. 0.01 ± 0.004; *P* < 0.01) than bulls in RCS pens. Slipping events and unsuccessful lie-down attempts occurred more frequently for bulls at the beginning than at the end of the fattening (0.35 ± 0.06 *v*. 0.21 ± 0.06 for slips; 0.08 ± 0.01 *v*. 0.04 ± 0.01 for attempts to lie down; *P* < 0.05). A significant breed × type of floor interaction (Figure 2) was recorded for the time required by bulls to lie down (*P* < 0.05). In particular, lying-down duration was longer for LIM bulls housed on CS floor, lower for CH and LIM bulls on RCS floor and intermediate for CH bulls on CS floor.

**Table 5:** Effect of the type of floor on behaviours of Charolais and Limousin bulls recorded during two 8-h observation sessions starting right after feed delivery carried out 1 month after the beginning and 2 weeks before the expected end of their finishing period in six commercial farms (least squares means)

Breed (B)	Charolais		Limousine			*P*-value		
Type of floor (TF)	CS	RCS	CS	RCS	SEM	B	TF	B × TF
Continuous behaviour (% of bulls)								
Standing	53.1	55.5	53.2	55.6	5.25	0.989	0.496	0.987
Lying posture								
Sternal recumbency	32.6	34.3	34.1	36.5	3.34	0.682	0.444	0.896
Sternal with one front limb extended	22.2	14.1	17.8	8.19	1.70	0.046	0.006	0.691
Sternal with two front limbs extended	1.36	1.24	0.71	0.75	0.38	0.157	0.939	0.872
Lateral recumbency	5.38	5.40	6.80	4.14	1.31	0.966	0.138	0.134
Resting	35.6	31.6	34.1	31.9	3.93	0.914	0.269	0.720
Eating	13.5	17.7	18.2	17.5	2.75	0.573	0.356	0.207
Ruminating								
While standing	5.14	4.62	3.97	3.44	0.76	0.308	0.300	0.993
While lying	11.3	12.9	12.8	12.5	1.96	0.854	0.557	0.413

CS = concrete slatted floor; RCS = rubber covered slatted floor; SEM = standard error of mean.

Charolais bulls housed on RCS floor had longer dorsal wall lengths of both front and hind claws compared to CH bulls on CS floor and LIM bulls on both floors (Table 6). Diagonal lengths of both front and hind claws were longer for CH bulls housed on RCS floor, shorter for LIM bulls housed on CS floor and intermediate for the other bulls. Toe angles measured on both front and hind claws were greater for LIM bulls kept in RCS pens, intermediate for LIM and CH bulls kept in CS pens and lower for CH bulls kept in RCS pens (Table 6).

**Table 6:** Effect of the type of floor on front and hind claw measurements of Charolais and Limousin bulls from six commercial farms at postmortem inspection (least squares means)

Breed (B)	Charolais		Limousine			*P*-value		
Type of floor (TF)	CS	RCS	CS	RCS	SEM	B	TF	B × TF
Number of bulls	37	36	24	24				
Front claws								
Dorsal wall length (cm)	7.58^c^	8.78^a^	7.68^c^	8.27^b^	0.11	0.076	<0.001	0.009
Diagonal length (cm)	17.6^b^	19.3^a^	16.5^c^	17.2^b^	0.16	<0.001	<0.001	0.001
Toe angle (°)	59.6^ab^	50.0^c^	55.0^b^	60.9^a^	1.46	0.059	0.164	<0.001
Hind claws								
Dorsal wall length (cm)	7.59^b^	8.86^a^	7.55^b^	8.01^b^	0.13	0.001	<0.001	0.002
Diagonal length (cm)	16.0^b^	17.4^a^	14.8^c^	15.4^bc^	0.17	<0.001	<0.001	0.018
Toe angle (°)	58.7^ab^	49.5^c^	54.5^b^	60.4^a^	1.20	0.012	0.135	<0.001

CS = concrete slatted floor; RCS = rubber covered slatted floor; SEM = standard error of mean.

a,b,c values within a row with different superscripts differ for the reported *P-*value.

## Discussion

Supporting the previous findings of Alberti *et al*. (2008), the great growth potential of CH bulls resulted in a heavier slaughter weight than LIM bulls. Bulls of both breeds showed improved growth performance on RCS floor, and this positive effect of rubberized concrete floor coverings was also confirmed by Cozzi *et al*. (2013). In literature, CH has been considered a sensitive breed to lameness, especially after a long-term housing on CS floor (Brscic *et al*., 2015a). In the present study, the rubber covering of concrete slats did not reduce the number of CH bulls that were early culled, but it tended only to lower the occurrence of bursitis. Results of our study showed that also LIM bulls suffered of the housing on hard surface like CS floor, increasing the occurrence of bursitis and the prevalence of animals treated for locomotor disorders. In beef cattle, leg lesions have been associated to the exposure of the limbs to hard and abrasive surfaces (Platz *et al*., 2007; Schulze Westerath *et al*., 2007), and the increased prevalence of bursitis observed in our study for both breeds on CS floor fully supports this finding. The occurrence of further integumental alterations such as lesion/wounds and hairless patches observed only in CH bulls might instead arise from additional stressors to their joints due to the heavier body weight which narrowed space allowance over time (Graunke *et al*., 2011; Wechsler, 2011; Elmore *et al*., 2015). Support to this assumption comes from Brscic *et al*. (2015a) who reported a higher occurrence of hairless patches and lesions/swellings in heavy bulls at the end of their finishing period. Absmanner *et al*. (2009) considered the extension of one front limb as a possible strategy to get some relief in the leg joints during lying. The reduced frequency of this posture observed in both breeds when kept in RCS pens could be a sign of their better comfort. The additional stress coming from the heavy BW of CH bulls might explain the significant breed effect observed for this lying posture. However, the reduced lying comfort was observed for all bulls as the fattening cycle progressed since the percentage of bulls in sternal recumbency decreased and that of those in lateral recumbency increased. Several authors (Platz *et al*., 2007; Graunke *et al*., 2011) hypothesized that the slippery surface of the concrete slatted floor was an explanation for the inhibition of social interactions involving powerful movements in beef cattle. Rubber covering for concrete floors has been shown to improve bulls’ confidence to exhibit natural behaviours and forceful social interactions such as head butt/displacements, chases and mounts, although it did not achieve the welfare potential of straw bedding (Lowe *et al*., 2001; Gygax *et al*., 2007a; Absmanner *et al*., 2009). In this study, the number of all these pen-mates’ interactions increased when LIM bulls were housed on the RCS floors. Charolais bulls showed a similar trend, except for the mounting events that did not change according to the type of floor. Mounting is a sexual behaviour shown by fattening bulls to establish the inner hierarchy among pen-mates (Phillips, 1993). The breed × type of floor interaction observed for this behaviour might have several explanations. When housed on a floor like RCS that provides a satisfactory hoof grip, the more excitable temperament of LIM breed, previously recorded by Phocas et al. (2006) and Hoppe *et al*. (2010), could have encouraged mounting events in an attempt to stabilize hierarchy among pen-mates. However, since it was demonstrated that finishing bulls change their behaviour in relation to space allowance (Gupta *et al*., 2007; Gygax *et al*., 2007b), we cannot exclude that the lower space allowance for CH bulls caused by their heavy BW and larger body frame might have inhibited the performing of extreme and more powerful social interaction as mounts, head butts/displacements or chases, especially when housing on hard surfaces. Considering that the observers in charge of the behavioural assessments were not blind to treatments, we could not fully exclude an additional observer effect although the study was implemented according to ethical conduct in research. In addition, in this study, bulls exercised more caution on movements as the fattening cycle progressed, and this finding may be supported by the decreasing number of slips and unsuccessful attempts to lie down recorded in the final part of fattening. However, it has been demonstrated that fattening bulls perform transitions more cautiously when kept on hard and slippery floors like CS because they are painful and potentially traumatic (Platz *et al*., 2007). Consistent with Gygax *et al*. (2007a), in our study, all bulls housed on RCS floor had a lower frequency either because of unsuccessful attempts to lie down or because of abnormal lying-down events, both well-known indicators of housing discomfort. The increased confidence towards the RCS floor was further supported by the shortest lying-down duration recorded on this type of floor. Bull cleanliness is an important hygienic and economic issue at slaughter, as extremely dirty animals could increase the risk of microbial contamination of carcass and meat (Lowe *et al*., 2001; Schulze Westerath *et al*., 2007). Research on flooring systems identified the drainage area and the floor material as the main factors affecting beef cattle cleanliness (Graunke *et al*., 2011). In our study, rubber covering reduced the drainage area of pens by 31%, but only the cleanliness of CH bulls was impaired. Based on literature (Gygax *et al*., 2007b), we hypothesize that the increased faecal output as a consequence of the heavy BW was mainly responsible for the worsened cleanliness of these animals. Moreover, since a dirty and wet coat might cause bull discomfort increasing the risk of skin lesions (Bosilevac *et al*., 2005), the impaired cleanliness of CH bulls could explain the prevalence of integumental alterations recorded on RCS floor.

It has been reported that, regardless of the type of flooring system, the claw condition of fattening bulls becomes worse with an increase in bulls’ BW (Stanek *et al*., 2004) and age (Fjeldaas *et al*., 2007). Moreover, the low abrasiveness of rubber flooring has shown to increase the occurrence of overgrown claws at the toe level resulting in longer dorsal wall and diagonal lengths compared to concrete flooring (Telezhenko *et al*., 2009). Our *postmortem* claw inspection clearly confirmed these findings suggesting further interesting outcomes. It is important to highlight that bull claws were never trimmed during fattening. Functional claw trimming is not a routine practice for beef cattle category due to the shortness of the fattening cycle and the high risk of injury for the trimmers. All bulls increased claws’ length when kept in RCS pens; however, a significant sharpening of the toe angle was observed only in the claws of CH bulls. These animals completed their finishing at a higher BW than LIM bulls, and the sharpening of their toe angle might have been a way to bear a greater weight load over a longer period. This growth-wear unbalance of the claw horn would cause the shifting of the weight bearing point to the bulbs area (Toussaint Raven, 1985), predisposing the claws to develop specific disorders on the sole (Kremer *et al*., 2007). On the other hand, even a prolonged housing of CH bulls on concrete slatted floor is supposed to lead their claws to the development of sole and white line lesions, since abrasive and hard surfaces provoke an extreme wear of the claw horn (Telezhenko *et al*., 2008; Graunke *et al*., 2011).

## Conclusion

On a perspective of the development of animal-friendly flooring systems tailor-made for specific beef cattle breeds, the use of RCS floor as an alternative to the CS floor could be advised for bulls like LIM that are finished at a lower final BW (around 600 kg) than CH bulls. The problem of hoof overgrowth of these animals recorded on RCS floor might be prevented by introducing a certain percentage of a more abrasive surface in the floor pen. Results of this study show that, despite the positive growth performance, health and welfare of CH bulls finished at a final BW above 700 kg were impaired by their housing on both concrete or rubberized slatted floors. Therefore, alternative flooring systems to the fully slatted floor should be tested by future studies in order to improve health and welfare of these heavy animals.

## Notes

L Magrin, 0000-0002-2153-6117

F Gottardo, 0000-0002-0427-2180

M Brscic, 0000-0001-8366-5961

B Contiero, 0000-0002-6586-5035

G Cozzi, 0000-0003-0408-1082

## Software and data repository resources

None of the data were deposited in an official repository.
